# Surgically treated fractures during pregnancy: fracture patterns, anesthetic strategy, radiation considerations, and maternal outcomes

**DOI:** 10.3389/fmed.2026.1885236

**Published:** 2026-08-18

**Authors:** Bahar Acemoǧlu Abul, Ayşenur Erbin, Ruken Abul Toz, Hüsnü Yılmaz, Engin Eceviz, Resul Karakuş, Mehmet Süleyman Abul

**Affiliations:** 1 Zeynep Kamil Kadin ve Cocuk Hastaliklari Egitim ve Arastirma Hastanesi, Istanbul, Türkiye; 2 TC Saglik Bakanligi Basaksehir Cam ve Sakura Sehir Hastanesi, Başakşehir, Türkiye; 3 TC Saglik Bakanligi Cermik Ilce Devlet Hastanesi, Çermik, Türkiye; 4 Kartal Dr Lutfi Kirdar Sehir Hastanesi, Istanbul, Türkiye; 5 Metin Sabanci Baltalimani Kemik Hastaliklari Egitim ve Arastirma Hastanesi, Istanbul, Türkiye

**Keywords:** anesthesia, fetal safety, fracture surgery, pregnancy, radiation safety

## Abstract

**Introduction:**

Fractures occurring during pregnancy pose unique challenges related to fetal safety, anesthesia management, and radiation exposure. This study aimed to evaluate fracture patterns, surgical strategies, anesthetic approaches, radiation exposure, and maternal–fetal outcomes in pregnant patients undergoing operative fracture treatment.

**Methods:**

A retrospective cohort study was conducted involving pregnant patients treated for fractures between 2014 and 2023. Of the 30 identified patients, 19 who underwent surgical treatment were included in the analysis. Patients managed conservatively (*n* = 9), those with insufficient clinical and radiological data (*n* = 1), and patients with pathological fractures (*n* = 1) were excluded. Demographic characteristics, gestational age at injury, mechanism of trauma, fracture type, anesthesia modality, fluoroscopy exposure, fracture union time, complications, and obstetric outcomes were analyzed.

**Results:**

The mean maternal age was 29.8 years, and the mean gestational age at the time of injury was 21.4 weeks. Lower extremity fractures were the most common, with ankle fractures being predominant. Regional anesthesia was administered in 63% of patients. The mean intraoperative fluoroscopy time was 17.8 ± 6.2 s. Fracture union was achieved in all patients. Based on available maternal and obstetric follow-up records, no complications attributable to anesthesia or radiation exposure were identified.

**Conclusion:**

Surgically treated fractures during pregnancy can be managed successfully through a multidisciplinary approach, appropriate anesthesia selection, and strict adherence to radiation safety protocols. Favorable short-term maternal and obstetric outcomes were observed in this cohort; however, these findings should be interpreted cautiously because standardized neonatal assessments and long-term neurodevelopmental follow-up were not available.

## Introduction

Pregnancy is associated with significant physiological and biomechanical changes that can influence injury patterns and the management of fractures. Increased ligamentous laxity, accentuated lumbar lordosis, a shifted center of gravity, weight gain, and alterations in bone metabolism may predispose pregnant women to musculoskeletal trauma, particularly fractures of the lower extremities (1, 2).

In the management of fractures during pregnancy, the fracture type, maternal functional requirements, gestational age, and potential risks to the fetus should be carefully considered. Delayed treatment or inadequate stabilization may result in prolonged immobilization, thromboembolic complications, malunion, and impaired maternal mobility, thereby adversely affecting both maternal and fetal health (3, 4). Although fractures during pregnancy are relatively uncommon, challenges such as minimizing radiation exposure, ensuring the safe use of anesthesia and medications for both the pregnant patient and the fetus, and determining appropriate surgical timing and treatment strategies can lead to uncertainty and concern for both patients and orthopedic surgeons (5, 6).

Advances in anesthetic techniques, improvements in radiation safety protocols, and the adoption of multidisciplinary care approaches have contributed to improved clinical outcomes in pregnant trauma patients (7). Nevertheless, the existing literature remains largely limited to small case series, and data regarding the outcomes of surgical treatment are still scarce.

This study aims to address this gap by evaluating fracture characteristics, treatment outcomes, anesthetic strategies, radiation exposure, and maternal–fetal outcomes in pregnant patients undergoing surgical treatment for fractures. We hypothesized that favorable maternal and obstetric outcomes can be achieved in surgically treated fractures during pregnancy when individualized surgical management and appropriate perioperative precautions are applied.

## Materials and methods

### Study design and population

This study was approved by the Institutional Ethics Committee of our hospital (Decision No: 2025/010.99/23/36) and conducted in accordance with the ethical principles of the Declaration of Helsinki.

**Figure 1:**
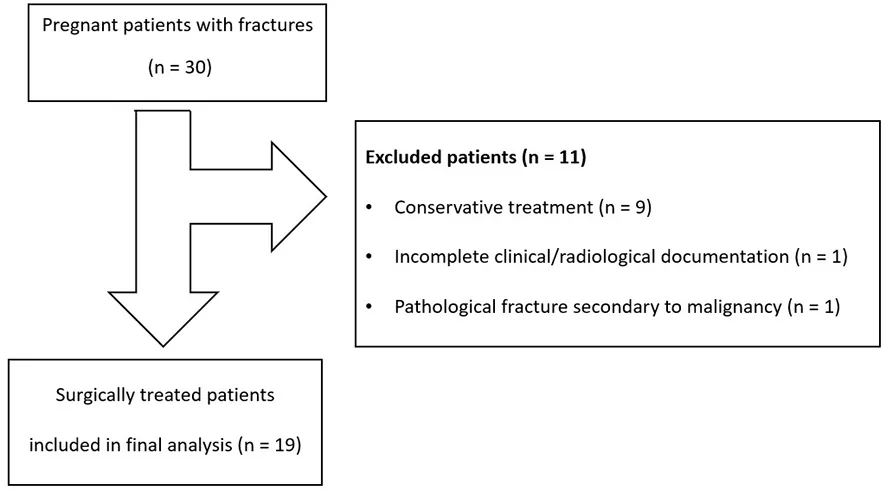
Flowchart of the patient selection process.

A total of 30 pregnant patients were initially identified. Patients with conservatively treated fractures (*n* = 9), insufficient clinical and radiological data (defined as incomplete documentation that prevented the reliable assessment of fracture characteristics, treatment details, or outcome measures; *n* = 1), and pathological fractures secondary to malignancy (*n* = 1) were excluded. Consequently, 19 patients who underwent surgical treatment were included in the final analysis (Figure 1).

### Data collection and variables

Maternal age, gestational age (GA) at the time of injury, the mechanism of trauma, fracture location, surgical procedure performed, type of anesthesia, the number of preoperative and intraoperative fluoroscopy exposures, cumulative intraoperative fluoroscopy time (defined as the total duration of all fluoroscopic exposures during each procedure), quality of reduction, time to union, postoperative complications, gestational age at delivery, delivery mode, birth weight, and Apgar score were retrospectively collected from electronic medical records. Maternal and fetal outcomes were obtained from hospital obstetric records and delivery data. Complete obstetric and neonatal outcome data through the time of delivery were available for all 19 included patients. Therefore, no handling of missing outcome data or imputation was required.

### Management protocols

Clinical management was carried out through multidisciplinary collaboration involving orthopedic surgeons, anesthesiologists, and obstetrics and gynecology specialists. All patients underwent preoperative obstetric consultation to assess maternal and fetal status, gestational age, and pregnancy-related considerations. Based on the obstetric evaluation, fracture characteristics, and surgical requirements, the anesthesiology team determined the most appropriate anesthetic approach. Surgical treatment was subsequently performed according to these multidisciplinary recommendations, with particular attention given to maternal safety and fetal protection. Fracture fixation was performed according to fracture location and morphology using standard orthopedic trauma techniques, including open reduction and internal fixation (ORIF), intramedullary nailing, and cannulated screw fixation, as appropriate for the fracture pattern. To minimize fetal exposure to anesthetic agents, regional anesthesia, consisting of spinal or epidural techniques, was administered whenever clinically feasible. In contrast, general anesthesia was preferred in cases involving complex fractures, pelvic or femoral fractures, or prolonged surgical procedures requiring airway control or specific patient positioning. Although patient-specific anesthetic agents and dosages were not consistently documented in the retrospective anesthesia records, routine anesthetic practice at our institution includes the use of bupivacaine or ropivacaine for spinal anesthesia and propofol induction, followed by maintenance with sevoflurane or desflurane for general anesthesia.

Radiological imaging and intraoperative fluoroscopy were minimized in accordance with the As Low As Reasonably Achievable (ALARA) principle. Radiation reduction strategies included limiting imaging to essential views only, minimizing fluoroscopy duration, using reduced fluoroscopy dose settings, limiting the number of fluoroscopy exposure counts whenever feasible, and routinely placing a 0.50-mm Pb-equivalent lead apron over the maternal abdomen to minimize fetal radiation exposure. Fetal and maternal dosimetry was not routinely performed. No patient underwent computed tomography (CT) during the preoperative diagnostic evaluation or postoperative follow-up; all diagnostic assessments were performed using plain radiographs only.

Postoperatively, patients underwent a repeat obstetric consultation, and further medical treatment, medication adjustments, and follow-up recommendations were provided according to the obstetric assessment and maternal–fetal status.

**Figure 2:**
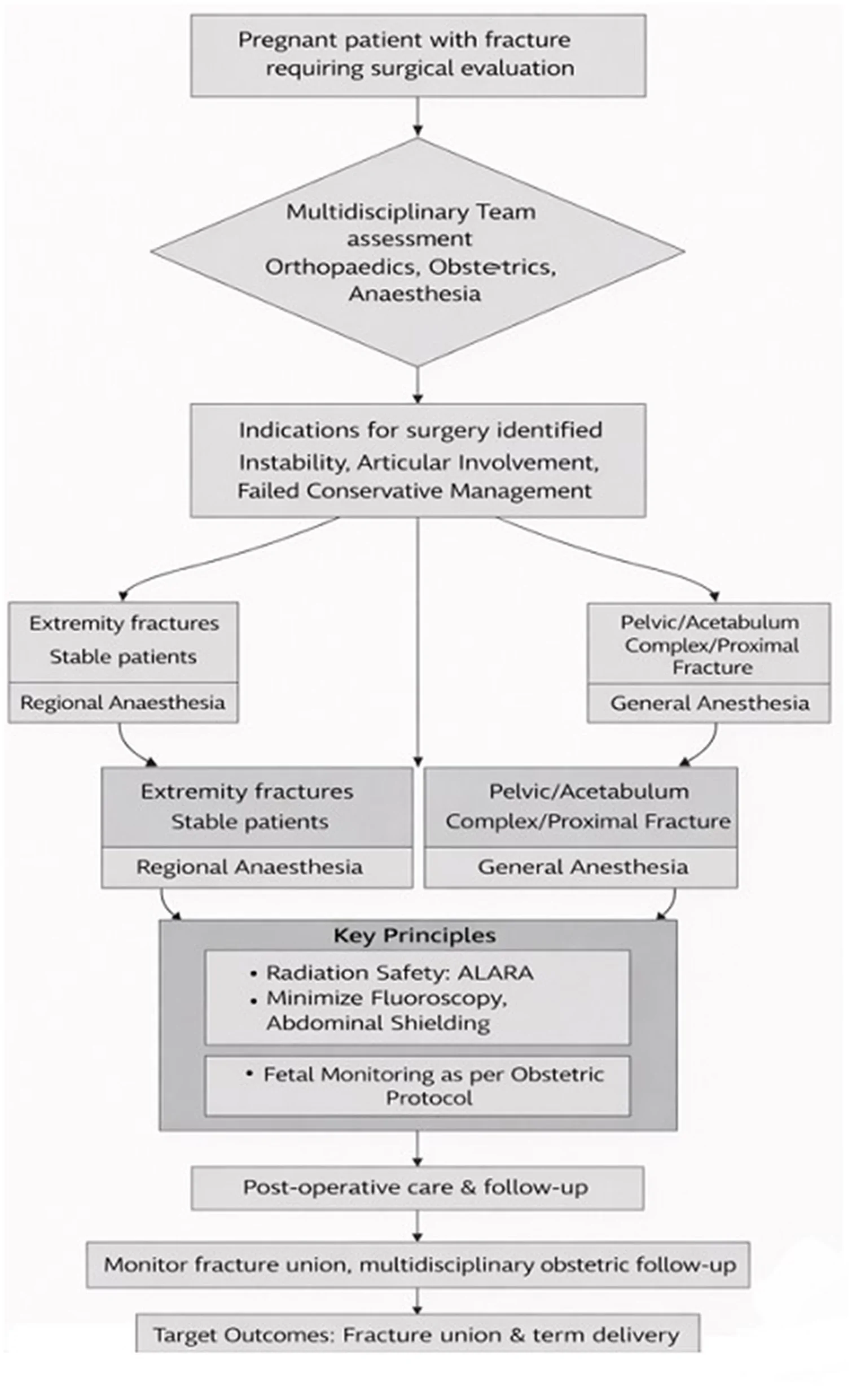
Flowchart of the clinical management pathway for surgically treated fractures during pregnancy.

Postoperative reduction quality was independently assessed on plain radiographs by two orthopedic surgeons who are experienced in trauma care using fracture-specific radiographic criteria reported in the orthopedic literature, including the restoration of alignment, acceptable residual displacement, and articular congruity (e.g., minimal articular step-off where applicable). Reduction quality was categorized as either good or poor. In cases of disagreement, the final classification was determined by consensus between the two assessors. Fracture union status and time to union were determined by reviewing radiographs obtained during outpatient follow-up visits after discharge. Radiographic evidence of callus formation across at least three cortices was considered indicative of fracture union. Maternal and fetal outcomes were assessed based on available obstetric follow-up records and delivery data reviewed by obstetrics and gynecology specialists. Standardized neonatal outcome measures and long-term neurodevelopmental follow-up were not routinely available due to the retrospective study design (Figure 2).

### Statistical analysis

All statistical analyses were performed using SPSS Statistics software (version 27.0, Armonk, NY, USA). The normality of continuous variables was assessed using the Shapiro–Wilk test. Descriptive statistics were used to summarize the data: continuous variables with a normal distribution were presented as mean ± standard deviation (SD), whereas non-normally distributed variables were presented as median (interquartile range [IQR]). Categorical variables were expressed as frequencies and percentages (*n*, %).

## Results

### Patient characteristics and fracture patterns

A total of 19 patients were included in the study. The mean maternal age was 29.8 ± 4.1 years, and the mean gestational age at the time of injury was 21.4 ± 6.5 weeks. The majority of injuries involved lower extremity fractures (84%, *n* = 16), with ankle (malleolar) fractures being the most common fracture type. Low-energy trauma mechanisms, such as twisting injuries and simple falls, were predominantly observed. The majority of patients sustained isolated orthopedic injuries, although a small number presented with multiple fractures. No associated non-orthopedic injuries requiring surgical intervention were identified. Patients were distributed across all pregnancy trimesters: 4 (21.1%) in the first trimester, 11 (57.9%) in the second trimester, and 4 (21.1%) in the third trimester. Owing to the limited number of patients in each subgroup, trimester-specific comparisons of maternal and neonatal outcomes were not performed (Table 1).

**Table 1:** Patient demographics, injury characteristics, and management details.

Pt.No	Age	GA	Trauma mechanism	Fracture location	Surgical procedure	Anesthesia	Fluoroscopy exposures	Reduction	Union time	Complication	Delivery week
1	33	32	Twisting	Tibia–fibula shaft	IM nail	Regional	4	Good	10 weeks	None	38
2	28	19	Twisting	Bimalleolar ankle	ORIF	Regional	3	Good	10 weeks	None	39
3	29	15	Twisting	Trimalleolar ankle	ORIF	Regional	4	Poor	12 weeks	None	38
4	32	24	Firearm	Talus	ORIF	General	3	Good	10 weeks	Superficial skin complication	41
5	26	13	Fall	Pilon	ORIF	Regional	4	Poor	10 weeks	Superficial skin complication	37
6	26	20	Twisting	Ankle	ORIF	Regional	2	Good	12 weeks	None	39
7	28	18	Fall	Humerus shaft	IM nail	General	5	Good	10 weeks	None	39
8	23	24	Suicide attempt	Pelvis + femur	Screws + IM	General	6	Good	8 weeks	None	40
9	35	25	Fall	Talus	ORIF	Regional	2	Poor	16 weeks	AVN	38
10	22	28	Assault	Forearm	ORIF	Regional	2	Good	12 weeks	None	39
11	35	8	Twisting	Trimalleolar	ORIF	Regional	3	Good	8 weeks	None	38
12	30	18	Assault	Metacarpals	ORIF	Regional	2	Good	8 weeks	None	41
13	33	24	Fall	Tibia–fibula	IM nail	General	6	Good	12 weeks	None	36
14	30	22	Fall	Distal humerus + radius	ORIF	General	6	Good	10 weeks	None	38
15	37	22	Twisting	Bimalleolar	ORIF	Regional	2	Good	10 weeks	None	38
16	22	32	Fall	Trimalleolar	ORIF	Regional	3	Good	12 weeks	None	40
17	32	13	Traffic	Femoral neck	Cannulated screws	General	5	Good	12 weeks	None	39
18	30	16	Fall	Pilon	ORIF	Regional	3	Poor	12 weeks	Compartment syndrome	39
19	29	15	Twisting	Trimalleolar	ORIF	Regional	3	Good	12 weeks	None	38

Pt. No., patient number; y, years; GA, gestational age; wk, week; IM, intramedullary; ORIF, open reduction and internal fixation; AVN, avascular necrosis.

### Anesthesia and surgical management

Regional anesthesia (spinal/epidural) was successfully used in 12 patients (63%). General anesthesia was administered in seven patients (37%) in cases involving polytrauma (pelvis + femur), proximal femur or humerus fractures, or complex upper extremity injuries. The mean number of preoperative radiographic images obtained per patient was 1.7 ± 0.8, whereas the mean number of intraoperative fluoroscopy exposures acquired per procedure was 3.6 ± 1.4. The mean cumulative intraoperative fluoroscopy time, representing the total duration of all fluoroscopic exposures during each procedure, was 17.8 ± 6.2 s (Table 2).

**Table 2:** Summary of cohort characteristics and outcomes.

Category	Value
Total pregnant patients with fractures	30
Surgically treated fractures (Study cohort)	19
Conservatively treated fractures (Excluded)	9
Patients with insufficient clinical and radiological data (Excluded)	1
Pathological fracture (Excluded)	1
Mean maternal age (years ± SD)	29.8 ± 4.1
Mean gestational age at injury (weeks ±SD)	21.4 ± 6.5
Predominant fracture region	Lower extremity [84% (16/19)]
Most common fracture type	Ankle region fractures (52.6%)
Predominant trauma mechanism	Low-energy (Twisting/Falls) (73.7%)
Primary anesthesia type	Regional [63% (12/19)]
Mean fluoroscopy exposures per Case (± SD)	3.6 ± 1.4
Cumulative intraoperative fluoroscopy time (seconds), mean ± SD	17.8 ±6.2
Mean fracture union time (weeks ± SD)	11.1 ± 2.0

### Outcomes

Fracture union was achieved in all patients, with a mean time to union of 11.1 ± 2.0 weeks. Postoperative reduction quality assessment demonstrated high inter-rater reliability between the two orthopedic surgeons, with an intraclass correlation coefficient (ICC) of 0.90. Postoperative complications were observed in four patients (21.1%), including two wound-related complications (wound dehiscence and superficial surgical site infection), one case of compartment syndrome, and one case of avascular necrosis of the talus following a severe talar fracture. No intraoperative anesthesia-related maternal complications were recorded (Table 2).

### Maternal–fetal outcomes

Based on available obstetric follow-up records, no fetal loss, congenital anomalies, or preterm deliveries were identified. The mean gestational age at delivery was 38.9 ± 1.2 weeks, indicating that the majority of patients delivered at term. Thirteen patients (68.4%) had vaginal deliveries, whereas six patients (31.6%) underwent cesarean section. The mean birth weight was 3,255 ± 452 g, and the mean Apgar score was 8.2 ± 0.4 (Table 3).

**Table 3:** Obstetric and neonatal outcomes.

Variable	Value
Maternal complications	4/19 (21.1%)
Fetal/obstetric complications	0
Mean delivery week (± SD)	38.9 ± 1.2
Delivery mode, *n* (%)	Vaginal: 13 (68.4%); Cesarean section: 6 (31.6%)
Birth weight (g), mean ± SD	3,255 ± 452 (2,680–4,050)
5-min Apgar score, mean ± SD	8.2 ± 0.4 (8–9)

## Discussion

This study presents a relatively large single-center case series focusing on fractures surgically treated during pregnancy. The majority of fractures were located in the lower extremities, particularly in the ankle (malleolar) region, and the trauma mechanisms were predominantly low-energy injuries. Regional anesthesia was successfully administered in most cases, whereas general anesthesia was preferred for patients with complex fracture patterns. Fracture union was achieved in all patients, the

complication rate was low, and no maternal or fetal complications related to surgical intervention, anesthesia, or radiation exposure were identified in the available clinical records. These findings suggest that favorable short-term orthopedic and maternal outcomes can be achieved when fractures during pregnancy are managed within a multidisciplinary framework and appropriate perioperative principles are applied.

In previous studies, fractures in pregnant patients have been reported to occur most frequently in weight-bearing joints, particularly the ankle and distal tibia regions (8). This pattern has been attributed to pregnancy-related biomechanical changes, including gestational weight gain, anterior displacement of the center of gravity, and ligamentous laxity, all of which can predispose patients to ankle injuries (9, 10). In addition, altered balance and gait mechanics during pregnancy have been shown to increase the risks of falls and low-energy trauma (11). In our study, similarly, the majority of injuries involved the lower extremities, with ankle and distal tibia fractures representing the most common fracture types. The predominance of twisting injuries and simple falls in our cohort is consistent with the proposed influence of pregnancy-related biomechanical and postural adaptations on this injury pattern. However, because body mass index (BMI) data were not consistently available in this retrospective cohort, the specific relationship between maternal body habitus and fracture patterns could not be evaluated.

The choice of anesthesia is a critical factor influencing clinical outcomes in pregnant orthopedic patients. The current orthopedic and anesthesiology literature recommends the use of regional anesthesia whenever feasible, particularly for extremity surgeries, because it better preserves maternal hemodynamic stability and reduces fetal exposure to anesthetic agents (5, 12–14, 19). In our series, regional anesthesia was successfully administered in the majority of patients with ankle and distal extremity fractures, which aligns with these recommendations. General anesthesia was preferred for complex injuries such as pelvic, femoral, or multiple fractures, particularly in cases requiring prolonged operative time and patient positioning that necessitated airway control, which reflects the practical considerations described in previous studies (12, 14). Based on the available clinical records, no anesthesia-related maternal complications or immediate fetal complications were identified. These findings suggest that individualized anesthetic selection based on fracture complexity, surgical requirements, and obstetric considerations is an appropriate approach to achieve favorable short-term maternal outcomes in pregnant orthopedic patients.

Radiation exposure during orthopedic trauma surgery in pregnancy remains one of the most debated issues. Fluoroscopy is frequently required for fracture reduction and implant positioning, particularly during intramedullary nailing and periarticular fixation procedures. The orthopedic literature consistently emphasizes adherence to the ALARA principle and the use of protective shielding to minimize fetal radiation exposure. Moreover, limited fluoroscopy use during extremity surgeries results in fetal radiation doses well below the thresholds associated with teratogenicity or fetal loss (6, 15). In our study, fluoroscopy exposure was deliberately minimized by implementing protective measures, with both preoperative and intraoperative imaging strictly limited. Based on the available clinical records, no early radiation-related fetal complications were identified. These findings suggest that, when appropriate radiation precautions are implemented, extremity surgeries can be performed while safely minimizing fetal radiation exposure. Furthermore, unlike many previous reports in the literature, our study quantitatively characterizes fluoroscopy use based on the number of fluoroscopy exposures and cumulative intraoperative fluoroscopy time, thereby providing a practical reference for orthopedic surgical practice.

Considering the physiological changes in calcium metabolism and bone turnover during pregnancy, fracture healing in pregnant patients remains a topic of clinical interest. Experimental and clinical data suggest that pregnancy does not adversely affect fracture healing and may even preserve or enhance bone remodeling capacity (1, 8). In our cohort, fracture union was achieved in all surgically treated patients, with a mean union time of 11.1 weeks, which is consistent with previous evidence suggesting that pregnancy does not adversely affect fracture healing. The observed complications, including superficial wound complications, avascular necrosis of the talus, and compartment syndrome, appeared to be attributable primarily to fracture severity and injury characteristics rather than pregnancy-specific physiological factors.

Another major concern in the management of orthopedic trauma during pregnancy is maternal and fetal outcomes. Previous studies have reported increased rates of adverse pregnancy outcomes following trauma, including preterm birth, placental abruption, poor neonatal condition at birth, perinatal mortality, and maternal mortality (7, 8, 16). However, these adverse outcomes have been reported predominantly in patients exposed to high-energy trauma or multisystem injuries rather than those with isolated orthopedic trauma (17, 18). In our cohort, the majority of injuries resulted from low-energy mechanisms, and a majority of the patients sustained isolated orthopedic injuries, which may explain the absence of fetal loss, congenital anomalies, or preterm delivery. The mean gestational age at delivery was 38.9 weeks, indicating that most pregnancies progressed to term. In addition, a majority of the patients delivered vaginally, and the observed birth weight and Apgar scores were within expected ranges for this cohort. Importantly, our cohort included patients who underwent surgical treatment across all three trimesters of pregnancy. Based on the available follow-up records, no adverse fetal outcomes were identified. However, given the limited sample size, particularly within trimester-specific subgroups, and the absence of standardized neonatal assessments and long-term neurodevelopmental follow-up, these findings should be interpreted cautiously. Overall, our findings suggest favorable short-term obstetric outcomes within this cohort but do not permit definitive conclusions regarding fetal safety beyond the immediate perinatal period (20).

This study has several limitations. Its retrospective case-series design, limited sample size, absence of a control group, and single-center setting may restrict the generalizability of the findings. Trimester-specific analyses were not feasible due to the limited sample size. In addition, BMI data were not consistently available, preventing the analysis of the potential association between maternal body habitus and fracture patterns. Although routine anesthetic practice at our institution was clarified, detailed patient-specific information regarding anesthetic agents and dosages was not consistently available owing to the retrospective study design. Although delivery mode, birth weight, and Apgar scores were available, standardized neonatal assessments and long-term neurodevelopmental follow-up were lacking because of the retrospective study design. Therefore, the present study cannot provide robust evidence regarding fetal safety beyond the immediate perinatal period. Direct radiation dose metrics, such as dose-area product (DAP), were also unavailable; therefore, although intraoperative fluoroscopy time was recorded, radiation exposure could not be directly quantified. Nevertheless, the study provides a well-defined cohort of surgically treated pregnant patients with detailed orthopedic outcome assessments, offering practical clinical data to inform the management of similar cases. Future multicenter prospective studies are warranted to strengthen the evidence.

## Conclusion

Fractures requiring surgical treatment during pregnancy can be managed successfully with a multidisciplinary approach. Individualized surgical planning, appropriate anesthesia selection, and adherence to radiation safety principles, including the ALARA concept and protective shielding, were applied throughout patient management in this cohort. Based on the available clinical and obstetric records, no fetal loss, congenital anomalies, or preterm deliveries were identified, and the observed neonatal outcomes were favorable. However, because standardized neonatal assessments and long-term neurodevelopmental follow-up were not available, these findings should not be interpreted as definitive evidence of fetal safety beyond the immediate perinatal period. Future prospective multicenter studies incorporating standardized neonatal outcome measures and long-term follow-up are warranted to further evaluate maternal and fetal outcomes.

## Data Availability

The raw data supporting the conclusions of this article will be made available by the authors, without undue reservation.
